# Silk Particles as Carriers of Therapeutic Molecules for Cancer Treatment

**DOI:** 10.3390/ma13214946

**Published:** 2020-11-04

**Authors:** Anna Florczak, Inga Grzechowiak, Tomasz Deptuch, Kamil Kucharczyk, Alicja Kaminska, Hanna Dams-Kozlowska

**Affiliations:** 1 Department of Medical Biotechnology, Poznan University of Medical Sciences, 61-866 Poznan, Poland; annaflorczak84@gmail.com (A.F.); grzechowiak.inga@gmail.com (I.G.); to.deptuch@gmail.com (T.D.); kamil.kucharczyk007@gmail.com (K.K.); stanislawska.alicja@gmail.com (A.K.); 2 Department of Diagnostics and Cancer Immunology, Greater Poland Cancer Centre, 61-866 Poznan, Poland

**Keywords:** silk, particles, cancer, silk fibroin, spidroin, sericin, bioengineering, drug delivery

## Abstract

Although progress is observed in cancer treatment, this disease continues to be the second leading cause of death worldwide. The current understanding of cancer indicates that treating cancer should not be limited to killing cancer cells alone, but that the target is the complex tumor microenvironment (TME). The application of nanoparticle-based drug delivery systems (DDS) can not only target cancer cells and TME, but also simultaneously resolve the severe side effects of various cancer treatment approaches, leading to more effective, precise, and less invasive therapy. Nanoparticles based on proteins derived from silkworms’ cocoons (like silk fibroin and sericins) and silk proteins from spiders (spidroins) are intensively explored not only in the oncology field. This natural-derived material offer biocompatibility, biodegradability, and simplicity of preparation methods. The protein-based material can be tailored for size, stability, drug loading/release kinetics, and functionalized with targeting ligands. This review summarizes the current status of drug delivery systems’ development based on proteins derived from silk fibroin, sericins, and spidroins, which application is focused on systemic cancer treatment. The nanoparticles that deliver chemotherapeutics, nucleic acid-based therapeutics, natural-derived agents, therapeutic proteins or peptides, inorganic compounds, as well as photosensitive molecules, are introduced.

## 1. Introduction

There are many types of cancer treatments. Depending on the type of cancer and how advanced it is, patients receive different treatments [1]. However, in most of the cases, a combinatory treatment is provided that may consist of surgery with chemotherapy and/or radiation therapy. Each of the indicated treatment methods is burdened with severe side effects. In recent years, a significant research effort had been made to develop a more effective, precise, and less invasive cancer treatment. Nanomedicine, immunotherapy, gene therapy, siRNA delivery, magnetic hyperthermia, thermal ablation, extracellular vesicles are currently extensively explored as alternative cancer therapy approaches [2,3,4,5].

Nanomedicine is based on the application of systems in which size is in the conventional range from 1 to 100 nm. However, the physicochemical and biological characteristics of the materials do not change abruptly at 100 nm; therefore, there is no bright line to set a maximum size limit [6]. Not only the size but other properties should be taken into account, including particle size distribution (PSD) and surface area (high surface-to-volume ratio) [6]. Nanoparticles, due to physicochemical properties, can encapsulate the significant quantity of active agents to increase their stability, biocompatibility, solubility in body fluids, and circulation time in an organism [7]. Thus, their use offers a great deal, especially in anti-cancer therapies, where the toxicity and other properties of chemotherapeutic drugs raise a significant challenge.

### 1.1. Nanoparticles in the Treatment of Cancer

Drug carriers for systemic cancer treatment have to overcome the challenges of reaching the tumor site and accumulating in the tumor microenvironment (TME). In general, nanoparticles (NPs) designed for intravenous administration and solid-tumor targeting must successfully exit the blood circulation at the tumor site, accumulate in the TME, target the tumor cells (or tumor-environmental cells), and trigger payload release [8]. Carriers designed for intracellular drug delivery must enter the target cells and complete their journey into the particular intracellular destination to achieve the desired outcome. Moreover, the carriers, upon the payload release, should undergo enzymatic degradation, and the biodegradation products should be well tolerated [9,10].

**Figure 1:**
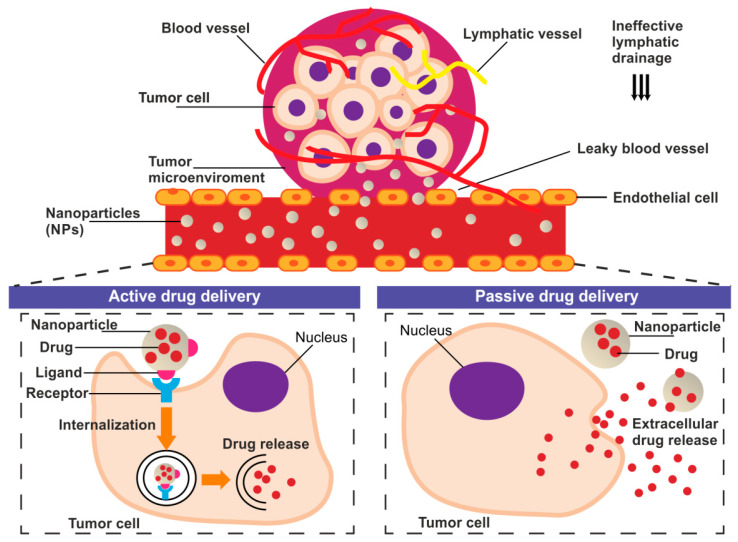
The schematic representation of the basic concept of the active and passive drug delivery systems in the tumor niche.

The tumor delivery of nanoparticles carrying chemotherapeutic agents might be provided through both passive and active targeting processes (Figure 1).

Passive drug targeting to solid tumor takes advantage of the enhanced permeability and retention (EPR) effect [11,12]. EPR exploit abnormalities of tumor vasculature manifested as hypervascularization, aberrant vascular architecture, and lack of lymphatic drainage [13]. The fenestrations in the vascular system allow the drug carriers accumulation in TME where enable prolonged, selective drug release [11,14]. However, the passive targeted accumulation of nanoparticles at TME is often insufficient to exert a drug effect [15]. For this reason, the translation of nanoparticle research into the clinic has been relatively slow and rather disappointing [16]. Studies over the last decade that used the nanoparticles showed that only approximately 0.7% of the administered nanomedicine dose reached the tumor [17]. The active targeting strategies are being proposed to challenge the limitations of passively-targeted nanoparticles [12]. Active tumor-targeting can be achieved by specific, ligand-mediated, cargo delivery to different types of objects: cancer cells or TME/TME-associated cells [18].

### 1.2. Silk as Material for Nanoparticles Preparation

Silk is a material whose application offers a lot of advantages, not only in nanomedicine. Silks are considered as biocompatible, biodegradable, non-toxic and they induce a mild immune response [19,20,21]. Although silk is protein-based material, it is characterized by the exceptional mechanical properties, compatibility with standard sterilization techniques (including high-temperature treatment), and simplicity of preparation methods [22,23,24,25]. Silk can be tailored for size, stability, drug loading/release kinetics by simply changing the process of the particle formation and post-treatment of the material [26]. Moreover, the active-targeting delivery may be achieved upon blending or conjugation of various targeting ligands such as peptides, antibodies, aptamers, etc. to the silk material. Such ligands can recognize and bound particular epitopes overexpressed on the surface of target cells [27,28]. Overall, the diverse silk fibroin (SF)-based materials show excellent bio-responses in vivo, including enzyme-dependent degradation and low immunogenicity [29]. Due to the ability to be degraded, silk fibroin structures can be remodeled in the body and replaced by native tissue [19]. The biocompatibility and biodegradation of silk biomaterials have been recently reviewed [19]. Furthermore, the intercellular degradation of silk nanoparticles was reported to be dependent on the enzymatic lysosomal function [30,31]. Thereby, the silk-based nanocarriers fulfill the critical requirement of carrier degradation and can be considered as safe drug delivery systems (DDSs) in vivo [32].

Silk is a fiber produced by the silkworm *Bombyx mori* (*B. mori*) to form its cocoon. It consists mainly of two proteins, fibroin, and sericin. Silk fibroin delivered from silkworm cocoons is the most commonly used silk for biomedical applications, including controlled drug delivery [33,34,35,36]. However, silk fibroins derived from other species of insects have also been investigated [36]. Additionally, proteins that coat the silk fibroin fiber, named sericins, are explored for biomedical applications [37]. Structures based on spider silks are another group of materials that are intensively researched in the field of medicine [38,39,40]. The mentioned above proteins can derive directly from the natural source (such as from cocoons, dragline silk from spiders) or can be produced biotechnologically [27,41,42]. Genetic engineering offers additional control over silk size, structure, targeting, and drug loading/release properties, among other characteristics [27,28,41].

**Figure 2:**
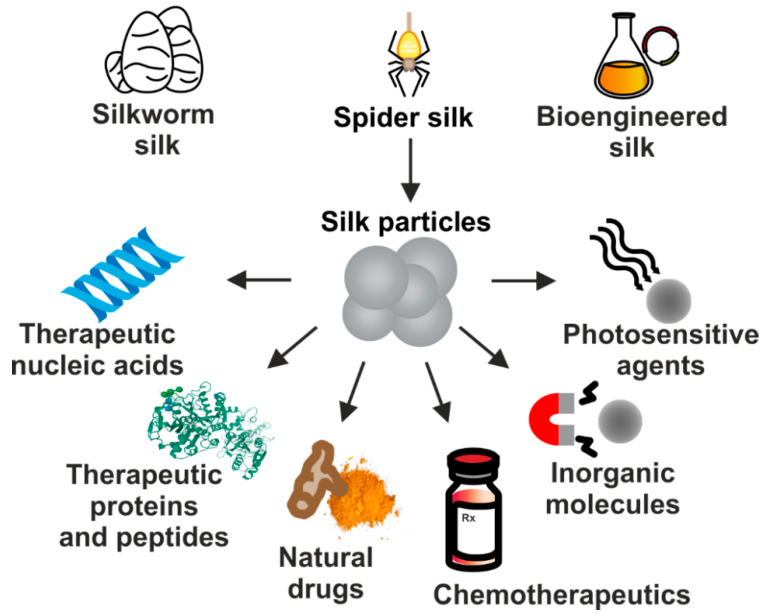
The proteins derived from natural silkworm cocoons, spider silks, and biotechnologically produced silk-derived proteins are used in the production of DDS for the systemic delivery of a variety of molecules used in cancer treatment.

Due to their excellent properties, silk-based carriers have been employed for the delivery of numerous therapeutic substances implementing different approaches to treat cancer. It includes particles carrying chemotherapeutics, nucleic acid-based therapeutics, plant-derived agents, therapeutic proteins or peptides, inorganic compounds, as well as photosensitive molecules (Figure 2). In this review, we summarize the current status of development of drug delivery systems that are based on proteins derived from silkworms’ cocoons and spidroins which application is focused on systemic cancer treatment.

## 2. Cancer Treatment Using Silk-Based Nanoparticles Loaded with Chemotherapeutics

Chemotherapy is often a first-line of the therapeutic approach for the treatment of a wide variety of cancers, as they are capable of combating cancer progression [43,44]. However, the effectiveness of the chemotherapeutic agents is limited in their clinical use due to the effects they exert on normal cells. Anti-cancer agents administered systemically at therapeutic doses may cause serious adverse events, including cardiotoxicity, hepatotoxicity, nephrotoxicity, gastrointestinal damage, or lung injury [45,46]. Accordingly, the therapeutic benefit-toxicity balance may not be acceptable for patients. DDSs can potentially resolve the problem of chemotherapeutic drug off-targeting effects. Silk-based nanoparticles have been extensively studied to deliver anti-cancer agents like paclitaxel, doxorubicin, floxuridine, methotrexate, etc. Entrapping a drug in the appropriate carrier increases a drug plasma retention time, tumor targeting, cellular uptake, and reduces the drug application frequency and systemic toxicity [47,48,49,50].

### 2.1. Doxorubicin

One of the most extensively studied chemotherapeutic agents for cancer treatment is an anthracycline drug, doxorubicin (Dox). Dox intercalates between base pairs in the DNA helix, thereby preventing DNA replication and ultimately inhibiting protein synthesis. Additionally, Dox inhibits topoisomerase II, blocking the resealing of the DNA double helix and thereby stopping the process of replication. Additional toxic effects of Dox include free radical damage, membrane perturbation, and apoptotic signaling [51]. The usefulness of Dox, however, is limited by considerable toxicity, especially damage to the cardiac muscle, which is cumulative and mostly irreversible, restricting extended use of this drug [51].

Dox-loaded *B. mori* silk fibroin nanoparticles (SFNPs) were examined in terms of a long-term response of human breast cancer cell lines [48]. The silk nanoparticles were prepared using an acetone precipitation method that allowed the formation of uniform (approximately 100 nm in diameter) and negatively charged nanoparticles. The obtained SFNPs were efficiently loaded with Dox (40 ng Dox/μg silk) and showed pH-dependent drug release. Dox release was investigated in different pH values that mimicked those of blood plasma (pH 7.4), early endosomes (pH 6.0), and lysosomes (pH 4.5). The drug release rate from SFNPs was enhanced significantly when Dox-loaded particles were incubated at pH 4.5 than for the other conditions. Seib et al. showed endocytic uptake and lysosomal accumulation of silk nanoparticles. In vitro studies with anthracycline-resistant MCF-7 breast cancer cells demonstrated that Dox-loaded silk nanoparticles were significantly more effective against cells compared with free Dox and were able to overcome drug resistance mechanisms [48].

Cao et al. investigated *B. mori* SFNPs produced in a one-step electrospraying method without emulsification of the components [52]. These nanoparticles were composed of SF, polyvinyl alcohol (PVA), and therapeutic drugs. The PVA/SF-NPs demonstrated a size range from 600 to 1800 nm. In this study, Dox was encapsulated in the PVA/SF-NPs with a greater than 90% encapsulation efficiency, and a controllable drug release profile was achieved by alternating the PVA/SF ratio. The higher PVA content in the PVA/SF-NPs led to smaller cumulative Dox release due to the denser molecular network of PVA. To accelerate the release of entrapped drug, the external ultrasound (US) stimuli was used. The application of US led to a 25% higher Dox release than from untreated particles. The PVA/SF-NPs efficiently induced apoptosis in MDA-MB-231 breast cancer cells, and the application of US enhanced significantly the cytotoxic effect of Dox delivered by PVA/SF-NPs. Additionally, PVA/SF-NPs elicited a pH-dependent drug release [52].

Most recently, *B. mori* silk/PVA particles were fabricated by using a co-flow microfluidic device [53]. The particles were formed through phase separation and self-association of silk when exposed to PVA, demonstrating a size in a range of 2.8–6.8 µm. Approximately 100% loading efficiency in the silk particles was obtained (41 µg Dox/mg of silk), and sustained Dox release occurred over 23 days. The cytotoxicity assay indicated the significant death of KELLY neuroblastoma cells and THP-1 macrophages. The internalization of silk particles into cells was demonstrated, and together with cytotoxicity results, it indicated that these silk particles might be utilized for drug delivery to multiple cell types within a TME [53].

To improve cancer targeting, the *B. mori*-derived SFNPs were conjugated with folic acid (FA) that provided specific recognition of cancer cells [54]. These particles were obtained by the salting-out method. Dox was grafted on the surface of these particles through chemical bonds (particles named FA-SFPs-DOX). In addition, Dox was physically encapsulated during the fabrication process of the silk fibroin particles (particles called FA-SFPs-DOX–DOX). Due to the double-loading strategy, the drug-loading capacity of the carriers was greatly improved. The double-loaded FA-SFPs-DOX–DOX particles were 530 nm in diameter and had a spherical porous structure. The pH-dependent drug release from FA-SFPs-DOX–DOX particles lasted for over 30 h. Moreover, the internalization of these particles was observed in cervical cancer cells (HeLa). The therapeutic ability of FA-conjugated particles was indicated, as FA-SFPs-DOX–DOX had significantly higher cytotoxicity against HeLa cells, compared with SFPs-DOX–DOX [54].

The further in vivo application of SFNPs can be limited by the relatively low colloidal stability, as they tend to aggregate in biological media. Several systems were designed to solve this potential problem, such as coating SFNPs with different cationic polymers, e.g., polyethylene glycol (PEG) [55,56,57,58], chitosan [59], polyethyleneimine (PEI) [60], and PEGylated PEI [61]. In a study by Wongpinyochit et al., Dox was encapsulated in PEGylated *B. mori* silk nanoparticles to achieve not only pH-controlled release of drugs but also to hide the particles from the reticuloendothelial system. Thanks to such design, the particles should remain in circulation for a more extended period [56]. The PEGylated silk fibroin-derived nanoparticles were generated using nanoprecipitation, demonstrating a size ~100 nm in diameter, negative zeta potential, excellent drug loading (>90%), and pH-dependent drug release capacity over 14 days. Dox-delivered by PEGylated silk nanoparticles induced greater cytotoxicity of MCF-7 breast cancer cells than freely diffusible chemotherapeutic. These particles also outperformed native silk nanoparticles for the Dox delivery to MCF-7 cells [56]. Further studies showed in a single-cell resolution that Dox-loaded native and PEGylated silk nanoparticles were efficiently endocytosed by MCF-7 cells [31]. Particles were trafficked to lysosomes, where a subsequent drug release was observed from the respective carriers. The released Dox localized in the nucleus within five h after particle internalization, inducing drug-associated cytotoxicity in vitro [31]. This study demonstrated the importance of lysosomal activity for drug release from silk nanoparticles [31].

In another anti-cancer DDS approach, *B. mori* silk fibroin has been used successfully to coat particles made of an amorphous calcium carbonate (ACC) core [62]. NPs were fabricated by coating Dox-preloaded ACC-DOX clusters with SF via a gas diffusion method. The size of the SF-ACC-DOX NPs was approximately 180 nm. The SF-ACC-DOX NPs targeted and were endocytosed into 4T1 murine breast cancer cells, as they were readily observed in the cytoplasm of the cells during two h incubation. The Dox released from cracked lysosomes was accumulated in the nucleus what resulted in the efficient killing of tumor cells. Moreover, the drug carrier SF-ACC NPs showed excellent biocompatibility in vitro. Additionally, the application of this drug carrier for Dox delivery drastically decreased the drug-associated side effects as compared to free drug administration in vivo [62].

Silk fibroin originated from *B. mori* mulberry silkworm has been the most extensively studied in the field of nanomedicine. However, the non-mulberry silk fibroin originated from Chinese temperate oak tasar silkworm *Antheraea pernyi* (ASF) was also investigated [63,64]. An ASF-based DDS system was developed by Lu et al. for Dox delivery [63]. Silk nanoparticles were obtained, taking advantage of the regenerated ASF sensitivity to temperature, which enabled conformational changes from α-helix to β-sheet in protein structure [63]. These ASF microspheres self-assembled at 35 °C, with the morphology and average size of the silk particles that depended on the pH. The nanoparticles were regular and spherical, with size in the range from 200 nm to 400 nm, when the pH value was below 4.3. The release rate of Dox from ASF particles was pH-dependent and sustained up to 23 days. However, the Dox-loading into ASF particles was relatively low (1.6%), and the efficiency of drug delivery was not studied in the cancer cells [63].

Wang et al. fabricated ASF nanoparticles by application of Na^+^, Ca^2+^, and Ce^3+^ ions to induce silk self-assembly. This method of NPs preparation provided their improved loading-release capacity of drugs [64]. These nanoparticles ranged in size from 100 to 500 nm. Dox was incorporated with high yield into ASF nanoparticles, evaluated as 0.14 mg Dox per mg of NPs. The release rate of this drug was pH-related. Moreover, the entrapped Dox in ASF particles induced a cytotoxic effect in HepG2 hepatoma cells [64].

The alternative silk fibroin-based DDS was elaborated by using the Indian tropical tasar silkworm *Antheraea mylitta* [65]. After particle formation using the desolvation technique, the carriers were conjugated with FA to achieve more effective tumor targeting. The silk fibroin FA-conjugated nanocarriers were less than 200 nm in diameter, non-toxic, and capable of sustained Dox release up to 21 days, with the enhanced release in the acidic pH. The Dox-loaded FA-silk NPs targeted MDA-MB-231cancer cells, were taken up by cells, and the delivered Dox significantly decreased the viability of cancer cells, as compared to non-functionalized vehicles [65].

**Figure 3:**
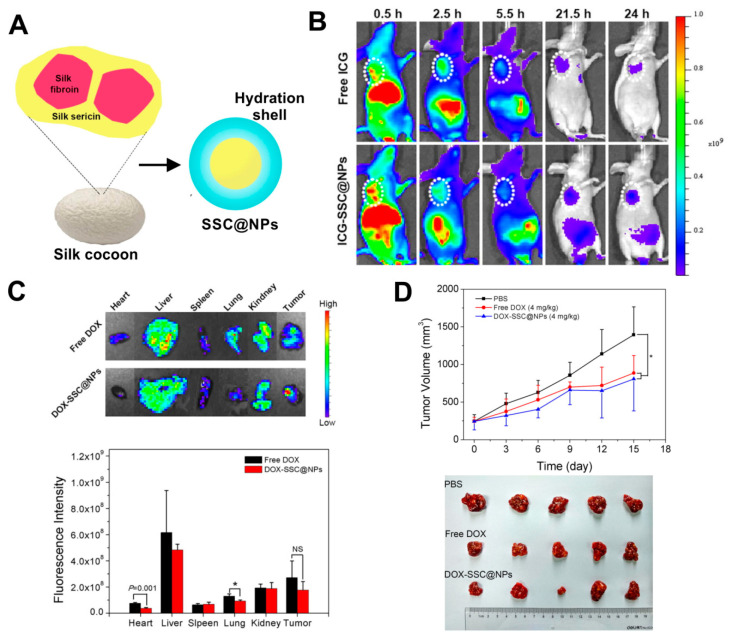
Hydrophilic silk sericin-based nanoparticles (SSC@NPs) in the treatment of cancer. (**A**) Schematic illustration of SSC@NPs. (**B**) The fluorescence imaging of mice bearing MCF-7 tumors at 24 h after intravenous administration of free dye indocyanine green ICG or ICG-SSC@NPs. The tumors are pointed with white circles. (**C**) Top: ex vivo images of major organs and tumors resected from HepG2-bearing mice after 24 h of treatment with free Dox and Dox-loaded DOX-SSC@NPs (NPs contained 2.5 mg/kg of Dox); Bottom: the graph demonstrating Dox distribution in major organs and tumors analyzed at 24 h post-injection. (**D**) In vivo therapeutic efficacy Dox-loaded DOX-SSC@NPs in reference to control Phosphate buffered saline PBS and free Dox in HepG2 tumor-bearing animals. Top: The time dependent tumor volumes after treatment. Bottom: Image of resected tumors. NS, not significant; * *p* < 0.05. Reproduced with permission [66]. Copyright, 2018, Elsevier.

Beside fibroin, also the other main component of a silkworm cocoon, silk sericin (SS), can be successfully used for nanoparticle formulation. Hu et al. implemented a two-step cross-linking of silk sericin derived from *B. mori* to chitosan, obtaining a sericin/chitosan-based nanoparticles (named SSC@NPs) with a size of approximately 200–300 nm [66]. The SSC@NPs were negatively charged in a neutral pH. However, in mildly acidic environment, the surface charge of SSC@NPs could undergo from negative to positive conversion [66]. Furthermore, due to its hydrophilicity, a water-soluble silk sericin showed both dispersion stabilizing and cryoprotective properties. Therefore, SSC@NPs displayed advantageous hydrophilicity, which resulted in improved colloidal stability of these particles in physiological fluids and their integrity during lyophilization. SSC@NPs were compatible in vitro with blood and when administrated intravenously in vivo. Also, Dox could be loaded with almost 50% encapsulation efficiency into SSC@NPs mainly by electrostatic interaction, and DOX-SSC@NPs displayed pH-dependent drug release profile. The DOX-SSC@NPs were indicated to mitigate the systemic toxicity of Dox in mice while maintaining the anti-tumor efficacy of the drug (Figure 3) [66].

*B. mori* silk sericin was also utilized to fabricate biocompatible and biodegradable micelles decorated with synthetic poly(c-benzyl-l-glutamate) (PBLG) via a ring-opening polymerization (ROP) strategy [35]. The introduction of PBLG chains onto sericin particles (SS-PBLG) significantly improved the micelle stability in aqueous solution. The mean diameter of the Dox-loaded SS-PBLG micelles was 110 nm. The micelles exhibited a drug loading efficiency of 13.8% and pH-responsive release of Dox. SS-PBLG-Dox provided excellent cell membrane penetration, resulting in the cellular delivery of Dox. A more efficient antitumor effect of drug delivered by SS-PBLG-Dox was induced in anthracycline drug-resistant (ADR) cell lines MCF-7/ADR and HepG2/ADR, both in vitro and in vivo, as compared to the same dose of a free chemotherapeutic [35]. Moreover, *A. pernyi* silk sericin (AS) has been used as a template to nucleate hydroxylapatite (HAp) nanoneedles to form porous sericin–HAp nanocomposite microspheres (ASMs) of approximately 1.2 µm in size [67]. The ASMs revealed a Dox encapsulation efficiency up to 62.6%, and a controllable, sustained, and pH-dependent rate of drug release due to the presence of pH-responsive HAp. The results of cellular uptake and intracellular Dox distribution implied that the ASM carriers efficiently released Dox inside HeLa cancer cells, inducing drug-associated toxicity [67].

In another approach, folate-conjugated *B. mori* silk sericin nanoparticles (FA-SND) were generated via self-assembly [68]. The FA-SND nanoparticles were spherical and displayed an average size of approximately 50 nm. These nanoparticles exhibited a negative surface charge allowing to reduce the non-specific clearance from circulation in vivo and were hemocompatible. Moreover, the acidic environment (pH 5.0) triggered a significant Dox release from FA-SND. These nanoparticles specifically targeted human oral epithelium carcinoma KB cells that are rich in folate-receptor. The FA-SND nanoparticles were endocytosed into lysosomes, where the acidic microenvironment promoted a rapid release of Dox that was then transported to nuclei exerting toxicity [68].

In one of the strategies for obtaining DDS, an aqueous solution of *B. mori* silk sericin was used to coat mesoporous silica nanoparticles (MSNs) [69]. To obtain sericin-coated particles (SMSNs), MSNs were modified by a series of chemical reactions between amine groups of sericin and aldehyde groups of MSNs, resulting in the sericin adsorption on the MSN’s surface [69]. The sizes of obtained SMSNs and DOX-loaded SMSNs (DOX@SMSNs) were approximately 110 nm and 120 nm, respectively. The sericin coating of MSNs aimed to prevent the premature leakage of the entrapped Dox from MSNs before the particles could reach the targeted cells [69]. Once reaching the tumor, sericin’s cell-adhesive property enhanced the uptake of SMSNs by the tumor cells that, in turn, were transported into perinuclear lysosomes. The lysosomal trafficking allowed to avoid drug efflux that is mediated by membrane-bound pumps. On the other hand, lysosomal acidity triggered the relaxation between sericin and MSNs, and lysosomal proteases deconstructed sericin shell. This double action of the acidic environment and enzymes led to Dox burst release and its nuclear trafficking, inducing significant tumor cells apoptosis. Such an approach could overcome the multidrug resistance (MDR) of cells. DOX@SMSNs demonstrated efficient killing of Dox-resistant cells in vitro and significantly reduced Dox-resistant MCF-7/ADR tumor-growth in vivo while alleviating the systemic toxicity of the treatment [69].

The recombinant silk proteins were also studied as a material for constructing drug delivery systems. Silk-elastin-like (SELP) copolymer was used to produce drug delivery carriers for cancer treatment [70]. The SELP copolymer (SE8Y, with silk to elastin ratio of 1:8) self-assembled in the presence of Dox into uniform micellar-like NPs with an average hydrodynamic diameter of 50 nm. The hydrophobic interactions between the drug and silk blocks triggered the formation of the SELP particles. The drug was loaded in the SELP nanoparticles, with an efficiency of approximately 6.5%. This study demonstrated significant uptake of the SELP NPs by the HeLa cells through endocytosis. The enzymatic degradation of the copolymeric bioengineered NPs occured inside the cells, what resulted in extended and sustained drug release. Consequently, Dox entrapped within SELP NPs demonstrated higher cytotoxicity than free Dox [70].

**Figure 4:**
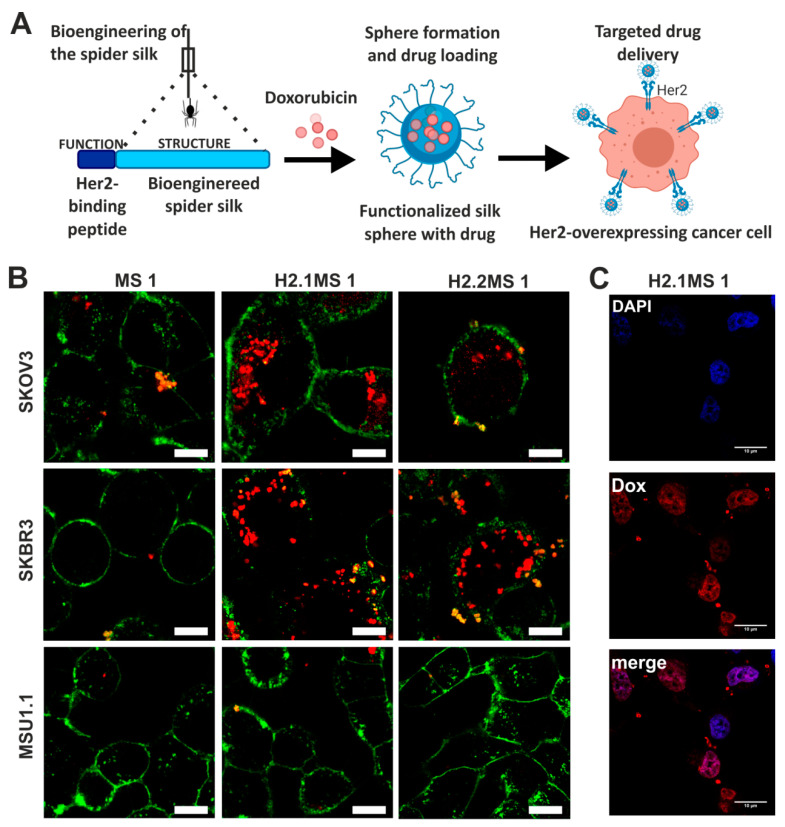
Functionalized spider silk nanospheres as drug carriers for targeted therapy of Her2-overexpressing tumors. (**A**) Schematic representation of DDS based on bioengineered spider silk that targets specifically Her2-overexpressing cancer cells. (**B**) Fluorescently labeled spheres that were functionalized with Her2-binding peptide H2.1 and H2.2 (H2.1MS1 and H2.2MS1) were effectively internalized into the cytoplasm of Her2(+) cells (SKOV-3 and SKBR-3) in contrast to non-functionalized spheres (MS1) and control Her2(−) (MSU1.1) cells. Cell membrane stained with ConA-FITC (green) and particles conjugated with ATTO 647N (red). Scale bar: 10 μm. (**C**) Dox was released from the H2.1MS1 spheres inside SKBR3 cells, and the colocalization of signals derived from Dox and the nucleus was observed. The nuclei stained with DAPI (blue) and autofluorescence of Dox (red). Scale bar: 10 μm. Reproduced with permission [71]. Copyright, 2014, American Chemical Society.

Doxorubicin was also delivered by nanoparticles made of bioengineered spider silk derived from *N. clavipes.* Florczak et al. developed the targeted drug delivery system based on the silk spheres MS1 derived from MaSp1 spidroin of *N. clavipes* spider that were functionalized with H2.1 and H2.2 peptides, recognizing Her2 molecule overexpressed on cancer cells (Figure 4) [71]. The silk spheres formed by salting-out with potassium phosphate, with sizes ranging from approximately 300 to 400 nm, were loaded with Dox with high incorporation efficiency. Dox content was approximately 350 ng per µg of spheres, and pH-dependent drug release up to 15 days was observed [71]. The functionalized nanoparticles efficiently delivered Dox to the cancer cells overexpressing Her2 (ovarian cancer cells SKOV-3 and breast cancer cells SKBR-3) what induced significantly higher cytotoxicity in these cells as compared with other cell lines without Her2-overexpression (MSU1.1 fibroblasts) and with control spheres without specific ligand [71]. The binding of the H2.1MS1 particles to Her2 molecule initialized the endocytosis process, and spheres were transported to endosomes and then to lysosomes [30]. Moreover, it was shown in vitro that spheres degradation occurred in the lysosomes due to the enzymatic activity and acidic environment [30]. Hence, Dox was delivered intracellularly (via H2.1MS1 particles), localized in nuclei (Figure 3), and killed the cells more efficiently than Dox released in the environment from non-targeted MS1 particles [71]. Accordingly, the developed system, based on the functionalization of silk with a ligand that targets tumor molecules, could increase the therapeutic index of chemotherapeutic agents against cancer cells. Simultaneously it could reduce the toxicity in healthy tissues. Similar results were obtained when the nanoparticles were prepared based on the blends of the two functionalized silk types (H2.1MS1 and H2.1MS2) [72]. MS2 is a bioengineered silk inspired on the Major ampullate Spidroin-2 (MaSp2) protein originated from *N. clavipes* spider [73]. The blending strategy was introduced to optimize the physical properties of the carriers, resulting in smaller (~250 nm in diameter), more uniform, spherical, and stable nanospheres [72]. Further improvement of these particle properties was achieved when the microfluidics system was implemented for their production. It allowed a more controlled manufacturing process of silk spheres to obtain particles of reproducible characteristics [74]. This DDS was additionally improved by double functionalization of bioengineered silk [75]. The functionalized MS2 silk containing a DOX peptide with an affinity for doxorubicin (DOXMS2) was blended with the H2.1MS1 silk that provides specificity towards cancer cells. These self-assembled nanoparticles (DOXMS2-H2.1MS1) showed a size below 400 nm in diameter, the improved control of Dox binding and release, and were taken-up explicitly by Her2-positive cancer cells (SKBR-3) [75]. Particles that release less Dox at neutral pH are critical to reducing the release of the drug into the circulation in vivo before the carrier reaches the tumor. It is of great importance in anti-cancer strategies development.

### 2.2. Cisplatin

Cisplatin is effective against various types of cancers, such as bladder, lung, ovarian, head and neck, and testicular cancers. Its mechanism of action is based on the cross-linking with the purine bases in the DNA, disrupting the mechanisms involved in DNA repair, It induces DNA damage, and finally resulting in cellular apoptosis [76]. However, the acquired drug resistance and numerous serious adverse events of cisplatin have led to the application of other platinum-containing anti-cancer drugs or platinum prodrugs.

Cisplatin-loaded *B. mori* silk fibroin nanoparticles were prepared by the electrospray method without using an organic solvent [47]. These particles were approximately 59 nm in diameter. The cisplatin-loaded silk fibroin nanoparticles showed enhanced cellular uptake by A-549 lung cancer cells, which caused the efficient toxicity to these cells. In contrast, the particles were not easily internalized into L-929 mouse fibroblasts, which resulted in weaker growth inhibition [47]. Another approach based on inhalable *B. mori* silk fibroin microparticles formulated by spray drying or spray-freeze-drying to target lung cancer was proposed by Kim et al. [77]. Cisplatin-incorporated silk carriers (of sizes ranging from ~10 to 22 µm) demonstrated the potential to deliver the drug directly to the lungs via dry powder inhalers. The optimized formulations of silk-based drug carriers were cross-linked by using genipin, and showed the increased extent of cisplatin release as compared to the particles without cross-linking. Hence, the cytotoxicity of cisplatin against A-549 lung cancer cells was demonstrated to be enhanced when delivered using the cross-linked silk based particles comparing with carriers without cross-linking [77].

Lozano-Perez et al. reported the preparation of *B. mori* SFNPs loaded with the hydrophobic platinum (IV) prodrug PtBz (PtBz-SFNPs). These particles, with a size of approximately 200 nm in diameter, were formed by rapid desolvation in polar organic solvents using high-power ultrasounds [78]. The obtained particles showed internalization into cells. PtBz–SFNPs triggered more substantial cytotoxic effects than free cisplatin and PtBz against human ovarian carcinoma A-2780 cells, their cisplatin-resistant variant A-2780cisR cells, and several breast tumor cell lines SKBR-3, MCF-7, and MDA-MB-231. The drug resistance to cisplatin was overcome. Furthermore, the cytotoxicity of PtBz–SFNs toward the non-tumorigenic renal cells LCC-PK1 was similar to that of cisplatin and less than that of PtBz [78].

### 2.3. Paclitaxel

Paclitaxel (PTX), a broad-spectrum anti-cancer drug, is widely used for the treatment of numerous types of cancers, mostly ovarian, lung, head and neck, breast, and Kaposi’s sarcoma [79]. PTX binds to tubulin and acts as microtubule stabilizer, thus impairing cell division and inducing apoptosis in cancer cells. However, low aqueous solubility and dose-limiting side effects, namely neurotoxicity and myelosuppression, limit significantly the effectiveness of PTX-based therapies [79]. PTX was successfully incorporated into silk fibroin nanoparticles in several studies [80,81,82,83,84]. Wu et al. presented PTX-loaded self-assembled *B. mori* silk fibroin nanoparticles (PTX-SFNPs) with a size of 130 nm in diameter [83]. The PTX-SFNPs were produced at room temperature in an aqueous solution by the desolvation method. In vitro studies on human gastric cancer cell lines, BGC-823 and SGC-7901 revealed the PTX-induced cytotoxicity when incorporated into PTX-SFNPs, while SFNPs showed no cytotoxicity towards cells [83]. Moreover, PTX-SFNPs demonstrated superior antitumor efficacy against gastric cancer in nude mice xenograft model by inhibiting tumor growth and diminishing tumor weights compared with free drug administrated systemically [83].

To further improve the efficacy of anti-cancer therapy, the multi-drug delivery platforms exploiting *B. mori* silk fibroin nanoparticles were investigated to achieve dual drug delivery [50]. The dual drug-loaded SF nanospheres were prepared using the desolvation method to deliver drugs that differed in physical property, such as hydrophilic Dox and hydrophobic PTX [50]. The size of the PTX/Dox-loaded nanospheres’ depended on concentrations of the SF and ethanol during the particle preparation process. By varying the ethanol concentration from 4% to 40% (*v*/*v*), the size of the PTX-loaded nanoparticles could be controlled in the range from 100 to 400 nm. Moreover, the increase of the initial SF concentration was correlated with increased PTX-loaded nanospheres’ size. The drug release of both hydrophobic PTX and hydrophilic Dox (adsorbed onto the surface of the nanospheres) from the particles was pH-dependent and sustained for over seven days. Further, the nanospheres loaded with binary drugs were effectively internalized via endocytosis and demonstrated more pronounced inhibition of HeLa and HepG2 cancer cell growth comparing with the nanospheres loaded with a single drug (Dox or PTX) or the same dose of free drugs [50].

As an alternative approach, a dual delivery system combining hydrogel with PTX and salinomycin-loaded *B. mori* SFNPs was designed by Wu et al. [84]. Salinomycin (Sal) has been known as an effective inhibitor of cancer stem cells (CSCs) and demonstrated antitumor efficacy in vivo [85]. The *B. mori* hydrogel was used as a carrier platform for PTX and Sal to kill CSCs and non-CSCs simultaneously. A resulting Sal-PTX-NP-Gel showed superior inhibition of tumor growth in vivo compared to the single drug-loaded hydrogel and systemic both free drugs administration in the murine hepatic carcinoma H22 tumor model. Sal-PTX-NP-Gel reduced the quantity of CD44+ and CD133+ cancer stem cells [84].

A novel kind of cancer cell targeting with anti-EGFR-iRGD nanobody conjugated to PTX-loaded *B. mori* silk fibroin nanoparticles was proposed by Bian et al. [80]. Epidermal growth factor receptor (EGFR) is overexpressed in multiple human solid tumors, and the iRGD is a cyclic, tumor-homing peptide that increases vascular and tissue permeability in a TME [80]. Therefore, such a design constitutes an interesting strategy for cancer therapy. For particle formation, PTX ethanolic solution was added dropwise into the aqueous SF solution inducing the self-assembly of SF. Subsequently, the NPs were conjugated with the nanobody (A-PTX-SF-NPs) by using a carbodiimide-mediated coupling procedure. These functionalized NPs showed a mean size of approximately 186 nm. The drug loading content was 10%, and drug encapsulation efficiency was 52%. These carriers targeted the EGFR-overexpressing tumor more effectively than NPs without functionalization, as observed in the imaging study. A-PTX-SF-NPs inhibited in vivo HeLa cancer cells with a high EGFR expression-in contrast to non-conjugated PTX-SF-NPs [80].

Aside from the most extensively studied mulberry cocoons of *B. mori*, a material derived from non-mulberry silk cocoon from *A. mylitta* was used for PTX delivery. The silk sericin extracted from these cocoons was blended with pluronic F-127 and F-87 in the presence of solvents to achieve self-assembled nanomicelles capable of carrying both hydrophilic and hydrophobic drugs [86]. The average size of obtained silk sericin-poloxamer (SS–P) NPs was ~100 nm in diameter that could be maintained during prolonged storage and incubation. The hydrophobic PTX was effectively encapsulated in self-assembled sericin-based nanoparticles (SS–P–P) [86]. The fabricated SS–P–P nanoparticles were not only stable in aqueous solution but also rapidly taken up by MCF-7 breast cancer cells. These drug-loaded nanoparticles showed efficient cytotoxicity towards MCF-7 cells when compared with the free drug [86].

### 2.4. 5′-Fluorouracil

5′-Fluorouracil (5-FU) is used in the treatment of a wide range of cancers, including breast, head and neck, pancreas, and cancers of the aerodigestive tract [87]. The mechanism of cytotoxicity of 5-FU has been ascribed to the misincorporation of fluoronucleotides into RNA and DNA. Moreover, it inhibits the action of thymidylate synthase (TS) that is a nucleotide synthesizing enzyme [87].

Several studies have reported encapsulated 5-FU in silk-based nanoparticles [88,89,90]. 5-FU-loaded SFNPs were prepared via nanoprecipitation with acetone, using different ratios of 5-FU and *B. mori* silk fibroin (1:1, 1:3, and 1:10, respectively) [90]. The optimal formulation was a ratio of 1:1, resulting in particle size of approximately 220 nm. These 5-FU-loaded SFNPs showed a high drug loading efficiency (~50%) with pH-dependent and sustained release of the drug *in vitro*. The cytotoxic effect of these nanoparticles against MCF-7 (human breast cancer) and HT-29 (human colon adenocarcinoma) cell lines was enhanced as compared to the free drug [90].

Reneeta et al. conjugated 5-FU to nanoparticles derived from silkworm *B. mori* pupal bio-waste [91]. Pupa-protein nanoparticles (PpNps) were prepared by the desolvation method, which resulted in a uniform particle size of approximately 160 nm. The drug entrapment efficiency and loading capacity of the developed 5-FU conjugated nanoparticles was 93% and 88.6%, respectively. 5FU-PpNps decreased the tumor cell viability in vitro and tumor volume in vivo, which proved its cytotoxic property towards cancer cells. Further, the reduced systemic toxicity was observed in 5FU-PpNps treated mice as compared with a free drug, manifested by a significant increase in total red blood cells (RBCs) and hemoglobulin with a decrease in white blood cells (WBCs) [91].

### 2.5. Floxuridine

Floxuridine (FUDR), a hydrophilic anti-cancer drug, is the pyrimidine analog with the antineoplastic activity that inhibits the TS. Its application results in disruption of DNA synthesis and cytotoxicity. FUDR is commonly used in the treatment of colon carcinoma and colorectal cancer. When FUDR is administrated by continuous infusion, direct anabolism to FUDR-monophosphate is enhanced, which increases the inhibition of DNA synthesis [92].

Yu et al. encapsulated FUDR in *B. mori* silk fibroin nanospheres prepared by using the self-assembly of silk protein via desolvation technique [93]. The average sizes of these SFNPs ranged from 210 to 510 nm [93]. The maximum drug loading reached about 6.8%, and the approximate drug release rate was two days. The FUDR-loaded nanospheres bounded to the HeLa cancer cells in contrast to the free FUDR, which was advantageous to elicit toxicity towards cancer cells. FUDR-loaded nanospheres killed more than 80% of HeLa cells after 24 h of incubation, implying their potential in future cancer treatments [93].

### 2.6. Methotrexate

Methotrexate (MTX) is a folate analog that inhibits folate-dependent synthetic reactions, leading to inhibition of DNA synthesis. It is used for several malignant and non-malignant diseases such as rheumatoid arthritis [94].

Subia et al. established the production of a nanoparticulate system composed of a blend of *B. mori* silk fibroin-albumin (SF–Alb) using a desolvation method [49]. The size of SF–Alb nanoparticles ranged from ~100 to 200 nm, and these particles were negatively charged, almost spherical, and stable. SF–Alb particles showed high loading efficiency of the poorly soluble MTX (87%, 85%, and 75% for 1:1, 2:1, and 1:2 SF–Alb blends, respectively) and sustained drug release. These nanoparticles were internalized by the MDA-MB-231 breast cancer cells, resided within the perinuclear area of cells [49]. In vitro study proved the effectiveness of MTX-SF–Alb nanoparticles in the killing of MDA-MB-231 cancerous breast cells but not the feline fibroblast AH-927 [49].

In a recent approach, Tallian et al. also developed a delivery system based on human serum albumin (HSA) and *B. mori* silk fibroin, using a desolvation method and an ultrasounds treatment [95]. These nanocapsules showed an average hydrodynamic radius between 438 and 888 nm and negative zeta potential. The pH-responsive MTX release depended on the SF concentration; the higher the SF content in the nanocapsules, the increased drug loading efficiency was observed. HSA-SF nanocapsules, composed of 50% SF, showed 98% encapsulation efficiency of MTX. An increased pH-responsive release of MTX at the acidic pH of 4 was indicated as compared with HSA particles without the silk component. Nanocapsules showed no significant toxic effect on the viability of monocytic THP-1 cells in concentration up to 62.5 µg/mL, suggesting its safety in future in vivo experiments [95].

### 2.7. Gemcitabine

Gemcitabine (Gem) is an FDA approved anti-cancer drug, which has a high potential in suppressing different carcinomas such as pancreas, bladder, and non-small cell carcinoma (NSCLC) [96]. The mechanisms of action of Gem involve inhibition of ribonucleotide reductase, which is needed for DNA synthesis, incorporation into DNA that halts the DNA synthesis, and induction of apoptosis through caspase signaling [97].

The *B. mori* silk fibroin particles were obtained by desolvation procedure and used for Gem incorporation [98]. These SFNPs were conjugated with SP5-52 peptide that provided specific targeting to NSCLC cells. The Gem-loaded SP5-52-conjugated SFNPs were 302 nm in diameter, with spherical morphology and negative surface charge, making it a suitable carrier for systemic drug delivery. Lung tumor-directed Gem-loaded SFNPs showed high cellular uptake, lysosomal localization, and caused a cytotoxic effect in vitro study. Further, their increased accumulation in the lung tissue in comparison with non-targeted SFNPs, as well as their improved therapeutic efficacy was observed in a mice model of Lewis lung (LL/2) tumor [98].

### 2.8. Other Chemotherapeutics (Etoposide, Mitoxantrone)

Spheres made of bioengineered spider silks MS1, MS2 and EMS2 derived from spidroins of *N. clavipes* spider also were investigated as carriers for other commonly used chemotherapeutic agents, etoposide and mitoxantrone [99,100]. Etoposide (Etp) is a podophyllotoxin-based drug that is widely applied for the treatment of lung, testicular tumors, lymphomas (Hodgkin’s and non-Hodgkin), leukemias, breast, and ovarian cancers [101]. It reacts with topoisomerase II and DNA, leading to the formation of the ternary complex, thereby preventing resealing of the opened strands of DNA double helix [101]. Mitoxantrone, a cytotoxic anthracenedione derivative, has been used in the treatment of various types of cancers, including breast and prostate cancers, and also in lymphomas, and leukemias [102]. Its mode of action has been linked to several different mechanisms. Mitoxantrone is a topoisomerase II inhibitor, it intercalates with DNA through hydrogen bonding and electrostatic interactions, and it causes DNA-protein cross-links. Finally, it affects the proliferation of immune cells, secretion of cytokines, prostaglandin biosynthesis, and calcium release [102].

The salting-out technique was implemented to produce the bioengineered MS1 and MS2 silk-based particles [99]. Jastrzebska et al. indicated that the differences in amino acid composition between the MS1 and MS2 silks determined the physical properties of silk particles. The MS1 and MS2 spheres, prepared at the silk concentration of 2.5 mg/mL using 2M potassium phosphate buffer (pH 8), differed significantly in sizes; the MS2 particles were smaller than MS1 particles (<800 nm vs. >1000 nm, respectively). Moreover, the primary and secondary structure of the silk proteins influenced the morphology and surface charge of the particles obtained, and thus the loading and release of drugs. Furthermore, the loading method, as well as the physicochemical properties of the incorporated drug, played a crucial role in the chemotherapeutics encapsulation efficiency. In the pre-loading method, a drug was incorporated during the silk carriers fabrication process. In contrast, in the diffusion-driven post-loading procedure, a drug solution was added after the formation of the spheres. The negatively-charged MS2 particles were a better choice for positively-charged mitoxantrone delivery than positively charged MS1 particles [99]. Additionally, the pre-loading method enabled higher loading and slower mitoxantrone release from MS2-based NPs, showing their greater applicability for the delivery of mitoxantrone than particles loaded using the diffusion-based method. However, the post-loaded MS2 particles were superior for neutral Etp delivery compared with pre-loaded MS2 NPs and MS1 spheres [99]. The MS2 protein was further modified by the addition of a glutamic acid residue within its amino acid sequence to obtain EMS2 silk [100]. Due to this alternation, the EMS2 particles possessed a more negative charge than MS2 spheres. EMS2 particles were obtained via the salting-out method and presented spherical morphology with a mean size of 530 nm. The modification of particle charge resulted in more efficient loading of Etp and mitoxantrone drugs into EMS2 than into MS2 spheres. The higher loading capacity and slower release rate indicated that EMS2 particles are a better choice to serve as a vehicle for Etp delivery than MS2 particles [100].

## 3. Cancer Treatment Using Silk-Based Nanoparticles Loaded with Natural Drugs

In recent years a significantly increased interest in research on cancer therapies using plant-derived therapeutics has been observed [103]. One of such substances is curcumin-a polyphenol isolated from *Curcuma longa* displaying anti-inflammatory and anti-tumorigenic properties [104]. The poor water solubility of curcumin results in its low bioavailability and limits the usage of this compound in cancer therapy. Multiple studies indicated that SFNPs could be used for curcumin delivery to cancer cells [59,88,105].

Gupta et al. investigated the anti-cancer effect of curcumin delivered to low (MCF-7) and high (MDA-MB-453) Her2/neu-expressing breast cancer cells using *B. mori* SF and fibroin/chitosan (SFCS) blended NPs [59]. The NPs were prepared using the devised capillary-microdot technique, and their size did not exceed 100 nm. NPs containing only SF provided the highest curcumin entrapment, release, and intracellular uptake into breast cancer cells as compared with NPs composed of the silk-chitosan blend. Moreover, the exposure to curcumin-loaded SF NPs caused a significant decrease in cell viability in both MCF-7 and MDA-MB-453 cells, contrary to SFCS-curcumin NPs treatment [59].

Li et al. proposed a strategy for dual drug loading into the *B. mori* silk fibroin nanoparticles [88]. SFNPs were prepared using the desolvation method and had an average particle size of 217 nm. The 5-FU in combination with curcumin were loaded into SFNPs, with a loading efficacy of 45% and 15% for 5-FU and curcumin, respectively. The bioavailability of both drugs was improved in comparison to free 5-FU and curcumin. The increased apoptosis of 4T1 breast cancer cells via the generation of cellular reactive oxygen species (ROS) was observed in vitro when the dual drug-loaded formulation was applied. Also, animal studies have shown a significant reduction of tumor size after intratumoral injection of the drugs-entrapped nanoparticles compared with free drug formulations [88].

A curcumin delivery system based on magnetic-silk core-shell nanoparticles (MSNPs) was developed by Song et al. [105]. The NPs were prepared with *B. mori* SF using the salting-out method and ranged from 90 to 350 nm in diameter. Tests performed on MDA-MB-231 breast cancer cells indicated that cellular uptake of curcumin encapsulated into MSNPs was significantly higher (up to 80%) than free curcumin. MDA-MB-231 cancer cells treated with MSNPs loaded with curcumin exhibited significantly lower viability than cells exposed only to MSNPs or free curcumin [105]. Moreover, the magnetic part of the particles provides the possibility of using an external magnet for cancer targeting.

Montalban et al. produced the *B. mori* SFNPs using the physical adsorption method (NPs with a mean size of 166 nm) and the coprecipitation method (NPs with a mean size of 171 nm) [106]. In vitro study using human hepatocellular carcinoma Hep3B cells and neuroblastoma KELLY cells treated with curcumin-loaded SFNPs resulted in a high decrease in cell viability. On the other hand, their cytotoxic effect observed in healthy human bone marrow-derived mesenchymal stem cells was low [106].

Xie et al. obtained similar results as indicated above, using HCT116 human colorectal cancer cells and NCM-460 normal colon mucosal epithelial cells [107]. The *B. mori* SF-curcumin NPs with a mean particle size below 100 nm were prepared using a method such as the solution-enhanced dispersion by supercritical CO_2_ (SEDS). After two days, curcumin-loaded NPs at the concentration higher than 10 μg/mL displayed a stronger anti-cancer effect towards HCT-116 cancer cells (>94%) than free cytotoxic drug 5-FU (~83%). Moreover, at the same concentration, the side effect of curcumin delivered by SFNPs was reduced comparting with the 5-FU application, as showed using normal NCM-460 colon cells [107].

Other plant-derived substances widely studied concerning cancer therapy include two terpenoids extracted from *Tripterygium wilfordii*–triptolide and celastrol, which both exhibit anti-cancer and anti-inflammation activities [108]. Similarly to curcumin, the clinical applications of these compounds are limited due to their low solubility in aqueous solutions [109].

The anti-cancer effects of celastrol and triptolide delivered by SF NPs were studied by Ding et al. [110]. The *B. mori* SFNPs were produced by the desolvation method, which allowed the formation of uniform particles with a mean diameter of 166 nm (NPs containing triptolide) and 170 nm (NPs containing celastrol). The NPs provided stable drug release profiles with cumulative triptolide and celastrol release, reaching 95% and 80% in 7 days, respectively. The treatment of MIA PaCA-2 and PANC-1 human pancreatic cancer cells with triptolide and celastrol-loaded NPs induced higher apoptosis rates compared to free drugs [110].

An increase in the cytotoxic effect of a plant-derived drug delivered by SFNPs was also demonstrated using Caco-2 colorectal and MCF-7 breast cancer cells [111]. Pham et al. formulated *B. mori* SF-based NPs (a mean size of 300 nm) containing α-mangostin using the desolvation method. The α-mangostin extracted from *Garcinia mangostana* has anti-tumor potential in breast, colon, and lung cancers. It was indicated that SF-α-mangostin-loaded NPs crosslinked with ethylcarbodiimide (EDC) or PEI displayed a more significant cytotoxic activity towards Caco-2 and MCF-7 cancer cells than free α-mangostin. They also provided a nearly threefold increase in the drug’s water solubility and a 90% reduction in hematotoxicity compared with free α-mangostin [111].

The SF might not only serve as a drug delivery agent itself, but it can also be successfully used to coat other particles. Cheema et al. studied the efficacy of *B. mori* silk fibroin coated liposomes that were loaded with a receptor kinase inhibitor of natural origin, emodin (SF-ELP) [112]. The SF-ELP significantly more suppressed the growth of MDA-MB-453 breast cancer cells compared with uncoated drug-loaded liposomes (ELP). Increased uptake of emodin delivered by SF-coated particles and its improved protection from quick release and metabolism contributed to a more extended availability of this drug to down-modulate of the Her2/neu pathways (PI3K, MAPK). It induced a higher breast cancer cell death as compared to emodin delivery via ELP [112].

Besides silk fibroin, silk sericin also appears to be a promising delivery agent for plant-derived substances in cancer therapy. Suktham et al. analyzed the effect of resveratrol-loaded sericin NPs against Caco-2 human colorectal adenocarcinoma cells and BJ skin fibroblasts [113]. The NPs were prepared using a solventless precipitation technique and ranged in size between 200–400 nm. Loading of resveratrol, a polyphenol with documented anti-carcinogenic and anti-inflammatory properties [114], into sericin-based NPs resulted in high encapsulation levels (71–75%) and intra-cellular internalization efficiency (97%) compared with free resveratrol. Moreover, they provided a sustained drug release profile for over 72 h. Drug-loaded NPs exhibited a cytotoxic effect on cancer cells while maintaining no cytotoxicity towards healthy cells [113].

A similar observation was made by Fuster et al. who assessed the viability of healthy cells and cancer cells exposed to SFNPs containing naringenin [115]. Naringenin, which is a natural flavonoid found in various fruits and vegetables, exhibits antioxidant, anti-inflammatory and anti-cancer properties. However, its instability and low water solubility limit the usage of free naringenin in cancer therapy. *B. mori* silk fibroin NPs were prepared using high power ultrasounds and desolvation method followed by naringenin adsorption to the SFNPs. The SFNPs loaded with the drug ranged in size from 157.6 to 180.1 nm. They were found to demonstrate a higher in vitro anti-cancer properties than free naringenin in HeLa cervical cancer cells. Moreover, the SFNPs containing naringenin significantly decreased cell viability in HeLa cells compared to healthy EA.hy926 umbilical immortalized cells [115].

## 4. Cancer Treatment Using Silk-Based Nanoparticles Loaded with Peptides and Proteins

The particle-like silk carriers can also bind and deliver peptides and proteins (e.g., enzymes, cytokines) [77,116,117,118,119,120,121]. The encapsulation of enzymes in the carriers may prolong their in vivo stability and help preserve their enzymatic activity over time. Blüm et al. tested the encapsulation efficiency of the model enzyme—β-galactosidase into the capsules made of eADF4(C16) bioengineered spider silk [117]. To induce the encapsulation, the aqueous solution of eADF4(C16) and β-galactosidase were mixed with silicon oil by vortexing. Then, obtained self-assembled capsules were transferred to the water/ethanol mixture to induce the β-sheet formation of the silk molecules. Encapsulation of the β-galactosidase in the silk vehicles allowed to preserve its enzymatic activity even in the presence of proteolytic enzymes [117]. Furthermore, the results of this proof-of-concept study suggested that it was possible to deliver active enzymes as well as their precursors or intermediates, which then can be activated upon the external trigger.

The ability to entrap enzyme in the silk particles without loss of its enzymatic activity was also reported by Cao et al. [118]. In the study, the silk fibroin solution obtained from *B. mori* cocoons was mixed with the β-glucosidase and then introduced rapidly into acetone. The obtained nanoparticles that entrapped the β-glucosidase had 50–150 nm in diameter and spherical morphology. The β-glucosidase-silk fibroin particles showed enzymatic activity similar to that of the native enzyme with only minor loss of the substrate affinity, proving that silk particles can be utilized as carriers for the enzymes [118].

Although the described above studies were not directly related to cancer treatment, they have proved that silk-based delivery systems can also be utilized as carriers for proteins or peptides showing anti-cancer properties. An example of such protein is lactoferrin. Bovine lactoferrin and its variants with different iron saturation levels (e.g., apo-bovine lactoferrin that is ~2% Fe saturated) were cytotoxic towards breast cancer MDA-MB-231 and MCF-7 cells in the in vitro studies [120]. In contrast, no such activity was reported towards control, non-cancer MCF-10-2A cells. In a study by Roy et al., lactoferrin and apo-bovine lactoferrin were encapsulated in the carriers prepared from the silk of non-mulberry Eri silkworms (*Samia cynthia ricini*). The particles were obtained through chopping, milling, and spray drying of the degummed Eri cocoons. The obtained particles showed round morphology with the diameter ranging from 150 to 250 nm (before the loading of lactoferrin) and 200 to 300 nm after the loading of lactoferrin [120]. The lactoferrin-loaded silk particles showed significantly higher internalization into the MDA-MB-231 and MCF-7 cells than the unloaded silk particles, whereas the apo-bovine lactoferrin showed significantly higher internalization only into the MCF-7 breast cancer cells. Also, both lactoferrin and apo-bovine lactoferrin loaded spheres induced cytotoxicity towards the tested cell lines in the in vitro studies, activating different apoptotic pathways depending on the EGFR status of the tested cell line [120].

Cytokines are used in immunotherapy to induce patients’ immune response against cancer cells [122]. The drawbacks of cytokine immunotherapy relate to their short in vivo stability and dose-dependent severe adverse side effects (i.e., flu-like reactions or vascular leak syndrome [123,124]). The application of cytokine carriers may resolve the problems mentioned above. In the context of bone regeneration, the possibility of utilizing silk spheres as carriers for the osteoinductive cytokines was presented by Bessa et al. [116]. In the study, particles produced from silk fibroin were used to transport bone morphogenetic proteins (BMPs), e.g., BMP-2, BMP-9, and BMP-14 [116]. After adding the BMPs to the soluble *B. mori* silk fibroin, the microparticles were obtained through the gradual addition of ethanol to the mixture. The obtained particles had a mean diameter of 2.7 µm and allowed for effective encapsulation of the BMPs and their sustained release up to 14 days [116]. Similarly, other cytokines could be loaded to the silk particles and used for cancer therapy.

Another often investigated approach in cancer therapy is related to the therapeutic peptide-based cancer vaccines [125]. The patients are immunized with short synthetic peptides derived from tumor-associated antigens. It stimulates the patient’s immune system to form a cellular response against the tumor antigens to eliminate cancer cells eventually [126]. However, peptide vaccines often induce low activation of T-cell response and offer limited therapeutic effects [127]. It might be due to their short in vivo stability caused by proteolytic degradation and fast clearance from the bloodstream. The silk carries can be possibly employed to effectively provide the bioavailability and stability of the peptide tumor vaccines. Lucke et al. analyzed the utility of particles made of eADF (C16) bioengineered spider silk as a carrier for the peptide vaccines [119]. The silk sequence was fused with a sequence of a model antigenic peptide derived from ovalbumin in the various configuration: (i) directly to the N-termini of silk, (ii) via the cathepsin B cleavable linker, or iii) via the cathepsin S cleavable linker [119]. The silk nanoparticles were produced by the salting-out method in the presence of 2 M potassium phosphate solution or 2–4 M ammonium sulfate solution. Depending on the analyzed variant of the spheres, their diameter ranged from 241 to 283 nm. In the in vitro studies, the particles were preferentially internalized by the bone marrow-derived dendritic cells, which belong to the antigen-presenting cells (APCs). Furthermore, the hybrid particles containing the cathepsin-cleavable linker were localized in the lysosomes, where cathepsin B and S effectively cleaved and released the transported ovalbumin peptide from the spheres. The silk spheres with antigenic ovalbumin peptide with cleavable linker-cathepsin S induced antigen-specific proliferation of cytotoxic T cells in both in vitro and in vivo studies without the adjuvant application [119]. Also, the response was specific to the antigenic peptide as the non-functionalized particles were not pro-inflammatory and did not induce unspecific immune responses [119]. Obtained results indicated that the silk particles could be effectively used as carriers for the peptide vaccines. In the future, the proposed strategy could be applied to generate therapeutic cancer vaccines.

## 5. Cancer Treatment Using Silk-Based Nanoparticles Loaded with Nucleic Acid-Based Therapeutics

Silk-originated NPs provide a suitable tool for the delivery of nucleic acid-based therapeutics. The application of silk-based NPs is a safer method of gene delivery than the usage of viral vectors, while still providing high transfection rates and possible target specificity [33]. Moreover, silk-based systems for delivery therapeutics such as siRNA, microRNA, antisense oligodeoxynucleotides (ASOs) also have been designed in recent years.

The delivery of anti-luciferase siRNA to H1299 human lung cancer cells using silk/oligochitosan-based NPs was studied by Shahbazi et al. [128]. The NPs were fabricated by the solvent/non-solvent method, and their size ranged from 250 nm to 450 nm. The addition of *B. mori* silk fibroin to the oligochitosan NPs enhanced loading capacity as well as the serum stability of nucleic acid. The in vitro silencing effect of luciferase was observed in stably expressing firefly luciferase H1299 cancer cells due to siRNA delivery by particles. The siRNA-loaded SF/oligochitosan NPs induced a higher silencing effect (up to 45%) compared with nucleic acid delivery by oligochitosan polyplexes (up to 32%) [128].

Song et al. used *B. mori* SF to produce magnetic-silk/PEI core-shell nanoparticles by the salting-out method [60]. The NPs ranged in size from 167–319 nm depending on the SF content and were used for targeted delivery of c-myc antisense oligodeoxynucleotides to MDA-MB-231 breast cancer cells. NPs containing SF exhibited lower surface charges and significantly reduced cytotoxicity compared with NPs coated only with PEI. The silk/PEI nanoparticles, with or without the magnetic part, were less toxic to human dermal fibroblasts (HDF) than to MDA-MB-231 cancer cells. Significantly higher uptake of oligodeoxynucleotides (over 70%) was achieved in a shorter time (within 20 min) using silk/PEI magnetic NPs and magnetofection in comparison with treating cells with the same type of NPs and without the presence of an external magnetic field [60].

An *A. pernyi* silk fibroin (ASF) particles were developed by Liu et al. for gene delivery to HCT-116 human colorectal carcinoma cells [129]. PEI/pDNA-GFP complexes were coated with arginyl-glycyl-aspartic acid (RGD) peptide-rich ASF via self-assembly, which resulted in the formation of NPs with a diameter of 231–365 nm. The presence of RGD peptide increased their target specificity in comparison with PEI/DNA binary complexes. Moreover, gene delivery using ASF-coated NPs resulted in higher post-transfection cells viability [129].

Not only silk fibroin but also the other main *B. mori* silkworm cocoon component-sericin was successfully used in the formulation of nanoparticles for nucleic acid delivery. Yalcin et al. investigated the delivery of siRNA into Hep-2 laryngeal cancer cells using albumin-sericin nanoparticles (Alb-Ser NPs) [130]. The Alb-Ser NPs had a mean size of 117 nm and were synthesized by the desolvation method. The particles were further functionalized by complexing with poly-l-lysine/siRNA and hyaluronic acid for targeted delivery to Hep-2 cells. NPs were loaded with siRNA targeting casein kinase 2 (CK2), Absent, Small, or Homeotic-Like (ASH2L), and Cyclin D1 (CCND1) genes, which are overexpressed in Hep-2 cells. The silencing effect achieved by individual administration of Alb-Ser NPs targeting the mentioned above genes was significantly higher (45–65%) compared with naked siRNA and resulted in significant cytotoxicity of targeted cells [130].

To achieve higher target specificity and transfection efficiency in cancer therapy, extensive research has been conducted using bioengineered silk derived from MaSp1 spidroin of *N. clavipes* spider [131]. Silk-based block copolymers genetically modified with poly-l-lysine and RGD sequences were used to prepare ionic complexes and transfect HeLa cervical cancer cells with pDNA encoding firefly luciferase. The complexes (mean diameter of 223 nm) with 30 lysine residues and 11 repeated RGD sequences were the most effective in terms of cell-binding and cellular pDNA uptake comparing with variants of the lower number of poly-l-lysine and RGD repeats [131]. High nucleic acid delivery efficiency was also achieved by adding a sequence encoding ppTG1, cell membrane destabilizing peptide, to the bioengineered silk modified with 30 lysine residues [132]. The transfection of HeLa cells with complexes (mean diameter of 99 nm) of silk-polylysine-ppTG1 dimers and pDNAs encoding GFP or firefly luciferase was higher comparing with silk-polylysine-ppTG1 monomers. The constant pDNA release from the complexes was observed to 144 h upon enzymatic silk degradation [132]. In another study, Numata et al. analyzed the delivery of pDNA encoding GFP using self-assembled silk complexes containing poly-l-lysine and tumor homing peptides (THP) such as F3 and Lyp1 [133]. F3 peptide shows target specificity towards nucleolin expressing tumor cells and endothelial cells, whereas Lyp1 peptide binds specifically to the p32 receptor overexpressed in various tumor-associated cells [134,135]. Complexes of approximately 237 nm in diameter made of the bioengineered silk containing 15 lysine residues and F3 THP sequences were the most promising vehicle for pDNA delivery into MDA-MB-435 melanoma cells and MDA-MB-231 breast cancer cells. F3 silk/poly-l-lysine complexes also exhibited lower cytotoxicity and higher target specificity than the nanocomplexes containing Lyp1 THP. Lyp1 was significantly cytotoxic towards non-tumorigenic mammary breast epithelial cells (MCF-10A) [133].

The cancer immunotherapy approach using bioengineered silk NPs that delivered therapeutic nucleic acid was investigated by Kozlowska et al. [136]. Bioengineered silk MS2 based on *N. clavipes* MaSp2 spidroin was genetically functionalized with a poly-lysine motif (15 lysine residues) for binding of nucleic acids (MS2KN). The MS2KN spheres were produced by the salting-out method and had a mean diameter of 202 nm. They efficiently bounded nucleic acid-based therapeutic CpG-*STAT3*siRNA what protected the oligonucleotides from degradation derived from serum nucleases. Due to the CpG molecule, the CpG-*STAT3*siRNA-loaded MS2KN spheres delivered cargo specifically to TLR9-positive immune cells. Application of MS2KN silk spheres not only increased the effectiveness of CpG-siRNA internalization into J774 macrophages but also prolonged its processing inside the cells as compared with free oligonucleotide therapeutic. It also resulted in the prolonged silencing of *STAT3* expression and significantly decreased the level of STAT3-regulated IL-6 mRNA in J774 macrophages (observed up to 72 h) [136]. The CpG-*STAT3*siRNA/MS2KN particles have great potential in the anti-cancer therapies targeting TME.

## 6. Cancer Treatment Using Silk-Based Nanoparticles Loaded with Inorganic Molecules

Silk can also be combined with metal compounds to increase their biocompatibility and applicability. The particles obtained from such composite material may offer advantages in both cancer diagnosis (e.g., magnetic resonance imaging (MRI)) and therapy (like targeted drug delivery, hyperthermia). The magnetic silk particles can be formed, in which iron oxide nanoparticles (IONPs) are incorporated in a silk biopolymer. Kucharczyk et al. obtained the composite spheres made of bioengineered spider silk EMS2 and IONPs using a salting-out method with potassium phosphate buffer [137]. The EMS2/IONPs particles had a mean diameter of 500 nm, and their magnetic properties were similar to plain IONPs. Moreover, the presence of IONPs resulted in more than a 2-fold increase in Dox loading efficiency and slower drug release at neutral pH as compared with EMS2 silk particles [137]. The minimal drug release at the pH of blood makes EMS2/IONPs spheres a more favorable for in vivo application.

The salting-out method with potassium phosphate buffer was also used for the production of composite spheres made of *B. mori* silk fibroin and IONPs [138]. The Fe_3_O_4_–SF microspheres exhibited magnetic properties, were able to bind and release Dox, and to accumulate in the cytoplasm of HeLa cervical cancer cells. The wide size distribution and large size of composite particles–up to 3000 nm, may be, however, a severe limitation for the in vivo application [138].

The study by Chen et al. demonstrated the production of magnetic silk particles by using the SEDS method [139]. The particles were composed of *B. mori* silk fibroin, IONPs, and chemotherapeutic drug methotrexate. The nanoparticles had a mean diameter of 75 nm and narrow size distribution. The skin permeation of MTX-Fe_3_O_4_-SF nanoparticles was significantly increased under the application of static and alternating magnetic fields (MF), which caused the higher transportation of MTX comparing with free drug and the particles with only one type of magnetic field applied [139].

**Figure 5:**
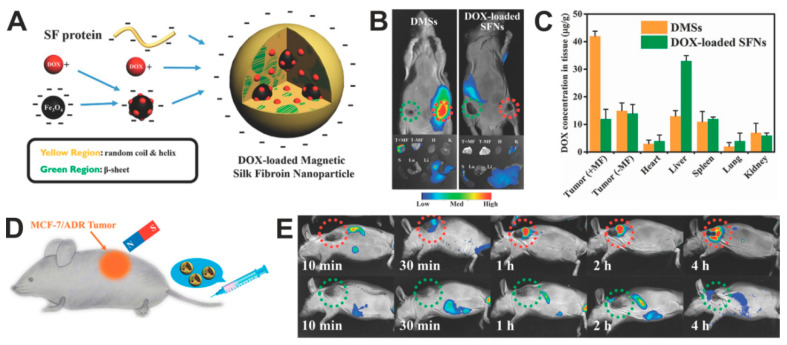
Doxorubicin-loaded magnetic silk fibroin nanoparticles (DMSs) in the targeted cancer treatment. (**A**) A scheme presenting the generation of DMSs: Dox is adsorbed in part on the surface of IONPs and then incorporated in the SF during the DMSs formation by using salting-out method. (**B**) Top panel: in vivo whole-body fluorescent imaging of MCF-7 breast cancer-bearing mice (tumors located on both sides) at 2 h after intravenous administration with DMSs or Dox-loaded SFNs; tumors location with and without magnet attachment are indicated with red and green circles, respectively. Bottom panel: tumors and major organs imaged ex vivo 12 h after intravenous administration of particles. (**C**) The biodistribution of the DMSs and Dox-loaded SFNs in the organs and tumors after particle administration and application of magnetic field at the tumor side; +MF and –MF: in the presence and absence of MF, respectively. (**D**) Schematic illustration of in vivo magnetic tumor targeting. (**E**) MCF-7/ADR tumors-bearing mice after injection of DMSs. DMSs contained 5 mg/kg of DOX. Reproduced with permission [140]. Copyright, 2014, Wiley.

The use of magnetic silk spheres for targeted in vivo drug delivery to the tumor was indicated by Tian et al. [140]. The spheres were made by the salting-out method with potassium phosphate buffer and consisted of *B. mori* silk fibroin, magnetic particles, and Dox. The particles had an average size of 130 nm and a relatively narrow PSD. The presence of IONPs enabled the magnetic guiding of composite particles and their accumulation in MCF7/ADR multidrug-resistant breast cancer (Figure 5). The targeted chemotherapy caused the inhibition of tumor growth and increased the survival of treated mice compared to the free Dox or administration of particles without MF application [140].

Kucharczyk et al. formed the composite spheres made of the blend of two functionalized bioengineered spider silks and IONPs [141]. One of the silks, H2.MS1 was modified with the peptide H2.1, which allowed specific targeting to Her2-overexpressing cancer cells [71,72]. The second silk was MS1Fe1 that contained a metal-binding peptide Fe1. The composite blended spheres were produced using the salting-out method with the potassium phosphate buffer. The particles made of functionalized silk MS1Fe1 demonstrated a higher affinity to IONPs than non-functionalized particles. The H2.1MS1:MS1Fe1/IONPs particles exhibited a specific affinity to Her2-overexpressing SKBR-3 breast cancer cells. Aside from the doxorubicin Dox delivery, the composite spheres generated heat upon MF treatment. The internalization of composite spheres into SKBR-3 breast cancer cells and subsequent administration of MF resulted in approximately six times higher levels of cell necrosis/late apoptosis than cells that did not receive particles. It indicated the potential of The H2.1MS1:MS1Fe1/IONPs spheres for hyperthermia therapy [141].

The quantum dot/silk composite system may find application in cell labeling, intracellular trafficking, and other imaging-related implementation in cancer diagnosis. Quantum dots coated by silk fibroin derived from *B. mori* (SF-coated QDs) were developed and characterized by Nathwani et al. [142,143]. The quantum dots were prepared by a one-pot synthesis method using CdO as a cadmium precursor and subsequently immersion in silk fibroin solution for coating. The SF-coated QDs were monodisperse with a size of approximately 10 nm. They were non-toxic against HeyA8 ovarian cancer cells and enabled their fluorescent in vitro imaging [142,143].

Chang et al. produced the silk-coated CdSe quantum dots (SF-CdSe QDs) via a one-step γ-radiation process [144]. This method was based on the γ-ray irradiation of the suspension containing components necessary for composite particle production, i.e., CdAc_2_, SeO_2_, (CH3)_2_CHOH, and *B. mori* silk fibroin water solution. The particles were approximately 5 nm in diameter and were internalized into the PANC-1 human pancreatic carcinoma cells. Moreover, the SF-CdSe QDs demonstrated much higher photostability under laser irradiation and much lower cytotoxicity against PANC-1 cells in comparison with free QDs [144].

The application of QD/silk for fluorescence imaging after in vivo implantation was evaluated by Zheng et al. [145]. In this study, QDs were incorporated in both silk microspheres and hydrogel. First, QDs were prepared by the synthesis in an aqueous medium using thiols as stabilizing agents. To form QD-incorporated silk microspheres, the obtained QDs were mixed with *B. mori* SF solution, and the PVA emulsification-drying method was used. For QD/silk hydrogel preparation, the QDs were mixed with silk solution, and then the ultrasonication was applied. Quantum dots were efficiently incorporated into both silk microspheres and hydrogel, which significantly reduced the toxicity of the QDs [145]. The QD/silk composites demonstrated strong fluorescence when subcutaneously injected in mice. The signal from QDs/silk microspheres was quenched within 24 h while fluorescence from the silk/QDs hydrogel remained stable up to 4 days after in vivo administration [145].

The simultaneous implementation of diagnostic imaging and treatment is the foundation of the theranostics concept developed not only for cancer therapy. Yang et al. indicated the application of silk-based composite material for theranostic purposes [146]. The silk fibroin obtained from *B. mori* cocoons was used to produce the nanoparticles loaded with fluorescent dye indocyanine green (ICG) and Dox. Additionally, the surface of the particles was mineralized by MnO_2_ (SF@MnO2/ICG/Dox) [146]. The method of their production consisted of silk fibroin bioinspired crystallization using KMnO_4_ and subsequent incubation with the ICG/Dox complex. The role of MnO_2_ is to generate O_2_ for photodynamic therapy (PDT) after the reaction with cancer cells metabolite H_2_O_2_. The remaining Mn2+ ions may serve as a contrast agent for MRI. ICG is a clinically approved photosensitizer. It is a photothermal adjuvant in hyperthermia treatment and enables the fluorescence visualization of cancer cells in vivo [147]. The SF@MnO_2_/ICG/Dox nanocarriers exhibited a hydrodynamic diameter of approximately 140 nm. When exposed to near-infrared (NIR) irradiation, they showed a strong and controllable photothermal response and accelerated drug release compared to Dox release without NIR application. The application of SF@MnO2/ICG/Dox particles and subsequent irradiation of 4T1 breast tumor-bearing mice resulted in a reduction of tumor size and higher survival of mice compared with animals treated with SF@MnO2/ICG/Dox particles without irradiation, or administration of free Dox or ICG with laser treatment [146].

## 7. Cancer Treatment Using Silk-Based Nanoparticles Loaded Photosensitive Agents

Phototherapy, represented by PDT and photothermal therapy (PTT), is considered a prospective therapeutic approach for cancer therapy with minimal invasion and high efficiency [148]. The PTT relies on using electromagnetic radiation. It uses light to heat photothermal agents (PT) to generate local hyperthermia and cause thermal damage in the tissue [149]. The PDT is related to the application of light absorbed by the photosensitizing agent (PS) and is used in conjunction with molecular oxygen [150]. After the PS excitation by the light of a specific wavelength, there are two possible pathways of interaction with the surroundings, named Type I and Type II reactions. The first type of interaction involves excited triplet state photosensitizer reaction with nucleic acids, proteins, and lipids through the hydrogen atoms transfer via a radical mechanism. As a result, free radicals and radical ions are generated, which, together with oxygen, cause the ROS formation [151]. In type II reactions, the triple-state excited PS reacts with oxygen in its triplet ground state. This phenomenon generates extremely reactive and cytotoxic singlet oxygen. Type I and II reactions take place simultaneously, and the balance between them depends on such aspects as the type of PS used or the oxygen concentration. The Type II pathway, resulting in singlet oxygen generation, is considered the major mechanism of photodynamic therapy [152].

The toxicity of administered and activated PS is mainly caused by the generation of ROS [153]. Their accumulation disturbs the structure and functions of cells and damages their biomolecules, leading to the release of inflammatory cytokines and chemokines. The death of cancer cells occurs primarily through necrosis and apoptosis. The apoptotic process is predominantly triggered by activation of two pathways–the death receptors or the mitochondrial permeabilization, both initiated by ROS. Moreover, the reactive oxygen species play a significant role in the induction of transcription factors/activators and genes associated with tumor suppression. Some of the molecular targets are hypoxia-inducible factor-1 alpha (HIF-1α), activator protein-1 (AP-1), signal transducer and activator of transcription 3 (STAT3), hedgehog protein (Hh), or nuclear factor kappa-light-chain-enhancer of activated B cells NF-κB [153]. However, it should be mention that the low level of ROS plays a vital role in the proliferation and homeostasis of cells and acts as a cellular signaling messenger. The normal cellular function depends on the preservation of the balance between the formation and elimination of ROS.

Various NIR substances have been utilized as agents for PDT and PTT. However, the hydrophobicity and photosensitivity of these molecules limit their further applications in biomedical fields. One possible approach to overcome these limitations is to encapsulate PS and PT agents in biopolymers.

*B. mori* SF-based NPs were prepared for embedding ICG to construct a therapeutic nano-platform (ICG-SFNPs) for PTT of glioblastoma [154]. The ICG was encapsulated into SFNPs with a high encapsulation efficiency during the SFNPs formation process via acetone precipitation. ICG-SFNPs exhibited a spherical morphology with a mean particle size of approximately 210 nm, negative zeta potential, and good stability in physiological medium. Compared with the ICG solution, ICG-SFNPs exhibited a more stable photothermal effect under the NIR irradiation and slow-release profile of ICG in vitro. Moreover, ICG-SFNPs were internalized into C6 glioma cells in vitro and effectively accumulated through the EPR effect inside the tumor site of C6 glioma-bearing xenograft nude mice. Finally, after two weeks of treatment, the tumor growth was significantly suppressed compared with the control group that received free ICG [154].

**Figure 6:**
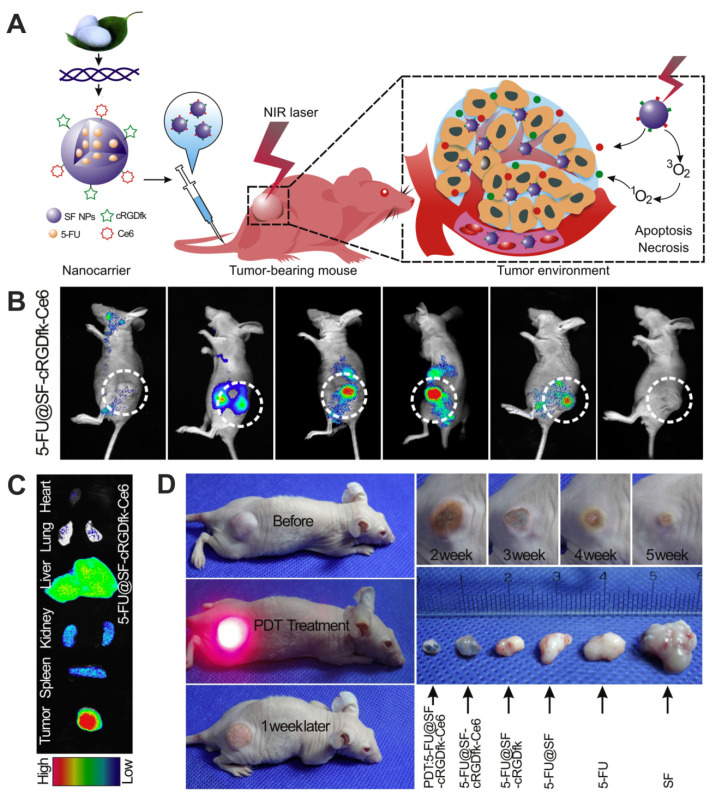
Functionalized silk fibroin nanoparticles for multimodal targeted chemotherapy and photodynamic therapy. (**A**) Schematic illustration of the SF-based nanocarriers functionalized with cyclic cRGDfk peptide, chlorin e6 and 5-FU for in vivo MGC-803 gastric cancer treatment. (**B**) In vivo dynamic fluorescence of 5-FU@SF-cRGDfk-Ce6 NPs injected to MGC-803 tumor-bearing mice (5-FU dose = 5 mg/kg). (**C**) Ex vivo fluorescent images of organs and tumor tissue 24 h post-injection of 5-FU@SF-cRGDfk-Ce6 NPs. (**D**) In vivo therapeutic efficacy of SF-based NPs and PDT. The tumor volumes after the treatment with PDT and 5-FU@SG-cRGDfk-Ce6 NPs in reference to the application of control SF-based NPs derivates and free 5-FU. Reproduced with permission [89]. Copyright, 2018, Elsevier.

The cyclic peptide cRGDfk and Chlorin e6 (Ce6) dye were conjugated with *B. mori* SF-based NPs (5-FU@SF-cRGDfk-Ce6) for combinational treatment of PDT and targeted delivery of 5-FU into gastric cancer (Figure 6) [89]. cRGDfk provides adhesion to cells, while Ce6 is a photosensitizer with antitumor activity when used in conjunction with irradiation. These particles were produced by using the acetone desolvation method, and then 5-FU-loading into SF-based NPs was achieved by chemical crosslinking with genipin. 5-FU@SF-cRGDfk-Ce6 particles were approximately 368 nm in diameter. The 5-FU@SF-cRGDfk-Ce6 NPs manifested sustained drug release, active tumor cell targeting, and excellent PDT potential in the αvβ3 integrin receptor-overexpressing MGC-803 gastric cancer cells in vitro. Moreover, the in vivo antitumor effect of these multifunctional SF-based NPs was evaluated in the gastric cancer xenograft mice model. Combined with laser irradiation, the 5-FU@SF-cRGDfk-Ce6 NPs reduced the tumor burden significantly in vivo as compared with mice treated with the 5-FU@SF-cRGDfk formulation or mice without laser irradiation (Figure 6) [89].

Besides silk fibroin, silk sericin originated from *B. mori* also appears to be a promising delivery platform for PT agents in cancer therapy. In a study by Deng et al., the hydrophilic sericin was modified with hydrophobic cholesterol to obtain an amphiphilic macromolecular conjugate (Ser-Chol) [155]. The tumor-targeting agent, folic acid, was further linked to the conjugate (FA-Ser-Chol) [155]. The organic solvent was used to induce self-assembly of the FA-Ser-Chol in the presence of the IR780 iodide (PS agent), resulting in the IR780 encapsulation and formation stable micelles (FA-Ser-Chol/IR780). The incorporation of IR780 into sericin-based micelles greatly improved its photostability and water solubility. These micelles (with a size of ~100 nm) could be efficiently absorbed by FA-positive gastric cancer cells (BGC-823) through FA receptors compared to micelles without a targeting ligand. Upon the cellular uptake and NIR laser irradiation, micelles showed remarkable PDT and PTT cytotoxicity towards BGC-823 cells [155].

Table 1 summarizes the different approaches for the use of silk-based drug delivery systems in the treatment of cancer.

## 8. Conclusions

A tumor, considered as an organ, demands comprehensive treatment to eliminate it effectively. Silk-based carriers have great potential for being used in this hard struggle. Most of the currently explored silk-derived DDSs take advantage of the EPR effect [35,48,50,52]. However, the effectiveness of silk spheres for actively-targeted drug delivery to tumors was also demonstrated, both in vitro [54,71] and in animal models [80,89]. The in vivo studies indicated that cellular recognition and sphere internalization greatly enhanced the toxic effect of the delivered drug [80,89]. However, the advantage of passive-delivery was successfully utilized in vivo for the deposition of photosensitive molecules in the tumor site [151]. One of the challenges for silk application in the clinic is silk heterogeneity. The described above silk-originated materials significantly differ in their amino acid sequence, morphology, and manufacturing process; thus, the results of their preclinical characterization cannot be directly translated between those studies. Although most of the studies concern the silkworm silk fibroin extracted from cocoons, the quality of the produced material depends on the environmental conditions. The recombinant production of the bioengineered silks enables to obtain a uniform type of silk. However, the protein expression in the heterologous host also has some obstacles such as LPS contamination, scale-up issue, and the silk size limitation. The preparation method also affects the properties of the silk particles (e.g., size, size distribution); it further complicates a direct comparison of the results for particles formed of the same silk type. Thus each potential application of silk particles needs careful study in physicochemical characterization, application effectiveness, toxicity, and immunogenicity.

The properties of native silk proteins plus, going a step ahead, also their modifications (due to blending, conjugation, or genetic engineering), offer the numerous potential combinations of silk and various active agents not only in the oncology field. The modification of silk is an open gate to explore. The blending of different silks is an exciting approach to obtain particles of new characteristics. The self-assembling silk proteins that follow similar rules independent of the silk origin allows forming spheres made of different types of silk. Moreover, blended silks can carry various functionalization. This functionalization can control the binding/release of cargo or binding to the cells. The blending strategy may allow to control of the number of cell-binding domains influencing the valence of the silk particles. The different multivalent silk spheres can be obtained by mixing the plain silk and silk functionalized with cell-binding peptide at various ratios. The particles that are weak or strong cell binders can be selected.

Summarizing, the properties of native silk proteins plus, going a step ahead, also their modifications (due to blending, conjugation, or genetic engineering), offer the numerous potential combinations of silk and various active agents not only in the oncology field. The increasing number of reports demonstrating the in vivo application of silk-based carriers indicate that delivery platforms based on proteins originated from silkworms cocoons and spider silks constitute an alternative but a realistic tool for cancer treatment.

## Figures and Tables

**Table 1:** Delivery of various anti-cancer therapeutic agents using silk-based nanoparticles.

Type of Anticancer Therapeutic	Therapeutic Agent	Silk Source	Particle Functionalization	Targeted Cancer	In Vitro Model	In Vivo Study	Reference
Chemo-therapeutic drugs	Doxorubicin	*B. mori* silk fibroin	-	Breast	MCF-7 and MCF-7-ADR	−	[48]
			-	Breast	MDA-MB-231	−	[55]
			-	Breast	MDA-MB-231	+	[52]
			-	Neuroblastoma	KELLY, THP-1	−	[53]
			FA	Cervical	HeLa	−	[54]
			PEG	Breast	MCF-7	−	[56]
		*A. pernyi* silk fibroin	-	Liver	HepG-2	−	[61]
		*A. mylitta* silk fibroin	FA	Breast	MDA-MB-231	−	[65]
		*B. mori* silk sericin-chitosan	-	Breast/Liver	MCF-7/HepG-2	+	[66]
		*A. pernyi* silk sericin	-	Breast/Cervical	Bcap-37/HeLa	−	[67]
		Bioengineered silk (SELP)	-	Cervical	HeLa	−	[70]
		Bioengineered *N. clavipes* spider silk (H2.1MS1, H2.2MS1)	H2.1 and H2.2 peptides (anti-Her2)	Breast/Ovarian	SKBR-3/SKOV-3	−	[30,71,72,74]
	Paclitaxel	*B. mori* silk fibroin	-	Gastric	BGC-823 SGC-7901	+	[83]
			-	Cervical/Liver	HeLa/HepG-2	−	[50]
			-	Pancreatic	CFPAC-1	−	[82]
			Anti-iRGD-EGFR	Cervical	HeLa	+	[80]
		*A. myllita* silk sericin	-	Breast	MCF-7	−	[86]
	Cisplatin	*B. mori* silk fibroin	-	Lung	A-549	−	[47,77]
			-	Ovarian/Breast	A-780, A-780-cisR/MCF-7, SKBR-3, MDA-MB-231	−	[78]
	5-Fluorouracil	*B. mori* silk fibroin	cRGDfk and Ce6	Gastric	MGC-803	+	[89]
			-	Breast	4T1	+	[88]
			-	Breast/Colon	MCF-7/HT-29	−	[90]
		*B. mori* pupa protein (Pp)	-	Lymphoma	DAL	+	[91]
	FUDR	*B. mori* silk fibroin	-	Cervical	HeLa	−	[93]
	Methotraxate	*B. mori* silk fibroin	-	ND	ND	+	[138]
		*B. mori* silk fibroin-albumin	-	Breast	MDA-MB-231	−	[49]
	Gemcitabine	*B. mori* silk fibroin	SP5-52 peptide	Lung	LL/2	+	[98]
Plant-derived therapeutic agents	Curcumin	*B. mori* silk fibroin	-	Liver/Neuroblastoma	Hep3B/KELLY	−	[106]
			-	Colon	HCT-116	−	[107]
			-	Breast	4T1	+	[88]
		*B. mori* silk fibroin-chitosan blend	-	Breast	MCF-7 and MDA-MB-453	−	[59]
	Resveratrol	*B. mori* silk sericin	-	Colon	Caco-2	−	[113]
	Triptolide/celastrol	*B. mori* silk fibroin	-	Pancreatic	MIA PaCA-2 and PANC-1	−	[110]
	Emodin	*B. mori* silk fibroin	-	Breast	BT-474, MCF-7and MDA-MB-453	−	[112]
	α-mangostin	*B. mori* silk fibroin	-	Colon/Breast	Caco-2/MCF-7	−	[111]
	Naringenin	*B. mori* silk fibroin	-	Cervical	HeLa	−	[115]
Nucleic acid-based therapeutic agents	siRNA (anti-LUC)	*B. mori* silk fibroin-oligochitosan blend	-	Lung	H1299	−	[128]
	pDNA encoding GFP	*A. pernyi* silk fibroin (ASF)	-	Colon	HCT-116	−	[129]
	siRNA (anti-CK2, anti-ASH2L, anti-Cyclin D1)	*B. mori* silk sericin-albumin	PLL and HA	Laryngeal	Hep-2	−	[130]
	siRNA (anti-STAT3)	Bioengineered *N. clavipes* spider silk (MS2KN)	PLL (KN)	ND	Macrophages J774	−	[136]
	pDNA encoding LUC	Bioengineered *N. clavipes* spider silk (15mer)	PLL and RGD	Cervical	HeLa	−	[131]
			PLL and ppTG1	Melanoma	MDA-MB-435	−	[132]
			PLL and Lyp1 or F3 peptide	Melanoma/Breast	MDA-MB-435/MDA-MB-231	+	[133]
Protein-based therapeutic agents	Lactoferrin	*S. cynthia ricini* Eri silk	-	Breast	MCF-7 and MDA-MB-231	−	[120]
	Peptides from ovoalbumin (C16-OVA)	Recombinant *A. diadematus* spider silk	-	ND	Bone marrow derived cells (BMDC)	+	[119]
Inorganic agents	IONPs/Dox	Bioengineered *N. clavipes* spider silk (MS1, MS2, EMS2)	H2.1 peptide (anti-Her2)	Breast	SKBR-3	−	[137,141]
	IONPs/Dox	*B. mori* silk fibroin	-	Breast	MCF-7 and MCF-7-ADR	+	[140]
	IONPs/Cur		-	Breast	MDA-MB-231	−	[105]
	IONPs/ODN (anti-c-myc)	*B. mori* silk fibroin mixed with PEI	-	Breast	MDA-MB-231	−	[60]
	MnO_2_/Dox/ICG	*B. mori* silk fibroin	-	Breast	4T1	+	[146]
Photo- sensitive agents	ICG	*B. mori* silk fibroin	-	Glioblastoma	C6	+	[151]
	Ce6/5-FU	*B. mori* silk fibroin	cRGDfk and Ce6	Gastric	MGC-803	+	[89]
	IR780	*B. mori* silk sericin-cholesterol	FA	Gastric	BGC-823	−	[152]

## Abbreviations

5-FU	5′-Fluorouracil
ADR	Anthracycline drug-resistant
Alb	Albumin
APC	Antigen-presenting cell
ASH2L	Absent, small or homeotic-like gene
ASO	Antisense oligodeoxynucleotide
CCND1	Cyclin D1 gene
Ce6	Chlorin e6
CK2	Casein kinase 2 gene
CSC	Cancer stem cell
DDS	Drug delivery system
Dox	Doxorubicin
eADF4(C16)	Bioengineered spider silk based on the ADF4 protein from *A. Diadematus* spider
EDC	Ethylcarbodiimide
EGFR	Epidermal growth factor receptor
EPR	Enhanced permeability and retention
Etp	Etoposide
FA	Folic acid
FUDR	Floxuridine
Gem	Gemcitabine
GFP	Green fluorescent protein
H2.1	Her2 recognizing peptide 1
H2.2	Her2 recognizing peptide 2
HAp	Hydroxyapatite
HSA	Human serum albumin
ICG	Indocyanine green
IONP	Iron oxide nanoparticle
MDR	Multi-drug resistance
MF	Magnetic field
MRI	Magnetic resonance imaging
MS1	Bioengineered silk based on the major ampullate spidroin-1 (masp1) protein from *N. Clavipes* spider
MS2	Bioengineered silk based on the major ampullate spidroin-2 (masp2) protein from *N. Clavipes* spider
MSN	Mesoporous silica nanoparticle
Mtn	Mitoxantrone
Mtx	Methotrexate
NIR	Near-infrared
NP	Nanoparticle
NSCLC	Non-small cell carcinoma
PBLG	Poly(c-benzyl-l-glutamate)
PBS	Phosphate buffered saline
PDT	Photodynamic therapy
PEG	Polyethylene glycol
PEI	Polyethyleneimine
PLL	Poly-l-Lysine
PS	Photosensitizing agent
PSD	Particle size distribution
PT	Photothermal agent
PTT	Photothermal therapy
PTX	Paclitaxel
PVA	Polyvinyl alcohol
QD	Quantum dot
RBC	Red blood cell
RGD	Arginyl-glycyl-aspartic acid peptide
ROS	Reactive oxygen species
Sal	Salinomycin
SEDS	Suspension-enhanced dispersion by supercritical CO_2_ method
SELP	Silk-elastin-like protein
SF	Silk fibroin
SFNP	Silk fibroin nanoparticle
SFP	Silk fibroin particle
SS	Silk sericin
THP	Tumor homing peptide
TME	Tumor microenvironment
TS	Thymidylatesynthetase
US	Ultrasound
WBC	White blood cell
